# Reduced Fractional Anisotropy of Corpus Callosum Modulates Inter-Hemispheric Resting State Functional Connectivity in Migraine Patients without Aura

**DOI:** 10.1371/journal.pone.0045476

**Published:** 2012-09-24

**Authors:** Kai Yuan, Wei Qin, Peng Liu, Ling Zhao, Dahua Yu, Limei Zhao, Minghao Dong, Jixin Liu, Xuejuan Yang, Karen M. von Deneen, Fanrong Liang, Jie Tian

**Affiliations:** 1 Life Sciences Research Center, School of Life Sciences and Technology, Xidian University, Xi’an, Shaanxi, China; 2 The 3rd Teaching Hospital, Chengdu University of Traditional Chinese Medicine, Chengdu, Sichuan, China; 3 Information Processing Laboratory, School of Information Engineering, Inner Mongolia University of Science and Technology, Baotou, Inner Mongolia, China; 4 Institute of Automation, Chinese Academy of Sciences, Beijing, China; Centre Hospitalier Universitaire Vaudois Lausanne - CHUV, UNIL, Switzerland

## Abstract

**Background:**

Diffusion tensor imaging (DTI) study revealed reduced fractional anisotropy (FA) values in the corpus callosum (CC) in migraine patients without aura. Abnormalities in white matter integrity, particularly in the CC, may affect inter-hemispheric resting state functional connectivity (RSFC). Unfortunately, relatively little is known about the alterations in functional interactions between the cerebral hemispheres during resting state in migraine patients without aura, and even less about how the inter-hemispheric RSFC are affected by the abnormalities of the CC.

**Methods and findings:**

Twenty-one migraine patients without aura and 21 healthy controls participated in this study, age-, sex-, and education-matched. Tract-based spatial statistics (TBSS) was employed to investigate the white matter alterations of the CC. Meanwhile, voxel-mirrored homotopic connectivity (VMHC) was used to compare the inter-hemispheric RSFC differences between the patients and controls. TBSS analysis revealed reduced FA values in the genu and the splenium of CC in patient group. VMHC analysis showed decreased inter-hemispheric RSFC of anterior cingulate cortex (ACC) in migraine patients without aura relative to that of the controls. Furthermore, in migraine patients without aura, the reduced FA values of the genu of CC correlated with the decreased inter-hemispheric RSFC of the ACC.

**Conclusions:**

Our findings demonstrated that the migraine patients without aura showed reduced FA values of the genu of CC and decreased inter-hemispheric RSFC of the ACC. The correlation between the above structural and functional changes suggested that the reduced fractional anisotropy (FA) of CC modulates inter-hemispheric VMHC in migraine patients without aura. Our results demonstrated that the VMHC alterations of ACC can reflect the FA changes of the genu of CC in migraine patients without aura.

## Introduction

Migraine is an idiopathic headache disorder, characterized as moderate to severe, often unilateral and pulsating headache attacks [1], [2]. With the advance in neuroimaging techniques, it has recently been regarded as a central nervous system disorder rather than merely a vascular, to a neurovascular disorder [1], [2]. Researchers have identified the probably migraine generator which is responsible for initiation of the migraine attack, i.e. the dorsal rostral pons [3], [4], [5], [6]. Apart from these lines, in patients with migraine structural [7], [8], [9], [10], [11] and functional [3], [4], [5], [6], [12], [13], [14] alterations have been observed in brain regions implicated with pain processing between migraine attacks, such as the anterior cingulate cortex (ACC), the orbitofrontal cortex (OFC), the insula, the temporal lobe, the posterior cingulate cortex (PCC), the supplementary motor area (SMA), the cerebellum and the thalamus. The reduced gray matter of the frontal lobes, brainstem, and the cerebellum were found to correlate with disease duration and severity, suggesting that more severe disease could result in more abnormalities that persist among migraines [1], [2], [15], [16].

Among previous findings, it is worthy to note that diffusion tensor imaging (DTI) method revealed the reduced fractional anisotropy (FA) values of the corpus callosum (CC) (genu, body, and splenium) in migraine patients without aura compared with the control group [17]. As the largest white matter structure in the brain, the CC is a wide bundle of neural fibers beneath the cortex in the brain at the longitudinal fissure, connecting the left and right cerebral hemispheres. The CC provides the main route of communication between the two hemispheres of the brain and facilitates inter-hemispheric communication [18], [19]. Abnormalities in white matter integrity, particularly in CC, may affect inter-hemispheric resting state functional connectivity (RSFC), which is fundamental to integrative attention processing and cognitive control [20], [21]. Unfortunately, relatively little is known about the alterations in functional interactions between the cerebral hemispheres during resting state in migraine patients without aura, and even less about the association between the inter-hemispheric RSFC changes and the abnormalities of the CC.

RSFC measures spontaneous brain activity of low-frequency fluctuations in blood oxygen level–dependent (BOLD) signals [22], which offers a route to directly quantify the inter-hemispheric functional interactions. During resting state, highly correlated spontaneous fluctuations are often observed within spatially separated but functionally related groups of cortical and subcortical regions, which consisted of the human brain’s intrinsic functional networks [23]. Vast majority of functional connections identified in these intrinsic functional networks are bilateral [24], [25]. Strong RSFC is even observable between homotopic regions with few monosynaptic callosal connections [26], [27], [28], which suggest that functional homotopy reflects an essential aspect of brain function [29]. Consistent with this conclusion, homotopic RSFC exhibits regional variation congruent with the brain’s functional hierarchy [30]. In addition, the developmental trajectories of homotopic RSFC show regional and hierarchical specificity across the life span [31], and homotopic RSFC is disrupted in autism [32] and cocaine addiction [33]. Therefore, homotopic RSFC may provide a sensitive indicator, which can reflect the effects of frequent nociceptive input on the pain processing circuits in migraine patients without aura.

To investigate that whether the abnormal white matter integrities of CC modulates inter-hemispheric RSFC in migraine patients without aura, multimodal MRI approach was employed in the current study. Firstly, tract-based spatial statistics (TBSS) was used to detect the FA differences of CC between the migraine patients and controls. Secondly, we examined inter-hemispheric RSFC in migraine patients without aura, using a novel approach, voxel-mirrored homotopic connectivity (VMHC) [31], [32], [33]. VMHC assesses the RSFC between each voxel and its mirrored counterpart in the other hemisphere. We compared VMHC differences between migraine patients without aura and controls and hypothesized that migraine patients would show abnormal inter-hemispheric RSFC. Thirdly, for the regions showed abnormal inter-hemispheric RSFC, seeding-based RSFC was employed to investigate the RSFC patterns within corresponding networks. Finally, we assessed the relationship between the white matter density of CC and abnormal inter-hemispheric RSFC in migraine patients without aura.

## Materials and Methods

This study was approved by the Medical Ethics Committee of the West China Hospital of Sichuan University and was conducted in accordance with the Declaration of Helsinki. All participants gave their written informed consent after the experimental procedure was fully explained.

### Participants

The diagnostic criteria of International Headache Society (IHS) for migraine without aura consists of the occurrence of at least 5 headache attacks that fulfill the following including criteria: 1) Headache attacks lasting 4–72 hours (untreated or unsuccessfully treated); 2) featuring at least two of the following characteristics: unilateral location, pulsating quality, moderate-to-severe pain intensity, and aggravation by causing avoidance of routine physical activity (e.g., walking or climbing stairs); 3) during the headache, they must have at least one of the following: nausea and/or vomiting, photophobia and phonophobia and 4) headache is not attributed to another disease [34]. According to the IHS criteria, migraine patients without aura were screened in our hospital. Twenty-one migraine patients without aura (16 females, aged 21–53 years, 32.4±10.3) were enrolled in our hospital. Patients rated the average pain intensity as 5.3±1.5 on a 0–10 scale derived from attacks in the past 4 weeks, with 10 being the most intense pain imaginable. In addition, twenty-one age- and gender-matched healthy controls (16 females, aged 22–54 years, 31.6±9.5) participated in our study. The controls either had no headache days per year or had family members who suffered regularly from a migraine or had other headaches. All of the participants were right-handed. Exclusion criteria for both groups were: 1) existence of a neurological disease; 2) alcohol, nicotine or drug abuse; 3) pregnancy or menstrual period in women; 4) any physical illness such as a brain tumor, hepatitis, or epilepsy as assessed according to clinical evaluations and medical records; and 5) claustrophobia. Patients were not having a migraine attack at least 72 hours prior to testing and no patient had a migraine precipitated during or on the day following the scan [12], [14]. The clinical characteristics of migraine patients without aura are shown in Table 1.

**Table 1:** Clinical information of migraine patients without aura and healthy controls (mean ± SD).

Items	migraine patients (N = 21)	healthy controls (N = 21)
*Age (years)*	32.4±10.3	31.6±9.5
*Gender (female/male)*	16/5	16/5
*Duration of disease (years)*	10.6±6.6	N/A
*Attack frequency (times)*	4.5±1.7	N/A
*Duration of a migraine attack (hours)*	13.4±5.8	N/A
*Average pain intensity (0–10)*	5.3±1.5	N/A

### Data Acquisitions

This experiment was carried out in a 3.0 Tesla Signa GE scanner with an 8-channel phase-array head coil at the Huaxi MR Research Center. For each subject, a high-resolution structural image was acquired by using a three-dimensional MRI sequence with a voxel size of 1 mm^3^ using an axial Fast Spoiled Gradient Recalled sequence (FSGPR) with the following parameters: repetition time (TR) = 1,900 ms; echo time (TE) = 2.26 ms; data matrix = 256×256; field of view (FOV) = 256 mm×256 mm. The resting-state functional images were obtained with an echo-planar imaging (EPI) with the following parameters: 30 contiguous slices with a slice thickness = 5 mm; TR = 2,000 ms; TE = 30 ms; flip angle = 90°; FOV = 240 mm×240 mm; data matrix = 64×64, and total volumes = 180. During the 6 minute functional scan, subjects were instructed to keep their eyes closed, not to think about anything, and to stay awake during the entire session. After the scan, the subjects were asked whether or not they remained awake during the whole procedure. Diffusion tensor images were acquired with 2 averages. The diffusion sensitizing gradients were applied along 30 non-linear directions (b = 1000 s/mm^2^) together with an acquisition without diffusion weighting (b = 0 s/mm^2^). The imaging parameters were 45 continuous axial slices with a slice thickness of 3 mm and no gap, FOV = 240 mm×240 mm; TR = 6800 ms; TE = 93 ms; data matrix = 128×128.

### DTI Data Analysis

DTI data preprocessing was carried out using FSL 4.1.9 (www.fmrib.ox.ac.uk/fsl/). First of all, correction for eddy-currents and head motion was done by means of affine registration on the first no-diffusion weighted volume of each subject. FA images were created by fitting the diffusion tensor to the raw diffusion data after brain extraction using BET [35]. Then, a voxel-wise statistical analysis of the FA data was carried out using the TBSS of FSL [36], [37]. FA images from all the subjects were realigned into an FMRIB58_FA standard-space image by FNIRT [38], [39] using a b-spline representation of the registration warp field [40]. The mean FA image was then created and thinned to create a mean FA skeleton (threshold of 0.2) representing the centers of all of the tracts common to the group. Each subject’s aligned FA data was then projected back onto this skeleton. Analysis of covariance (ANCOVA) was employed for statistical analysis, with age and gender effects as covariates. White matter FA value changes were assessed using permutation-based non-parametric testing [41] with 10000 random permutations. The threshold for significance was *p*<0.05, using threshold-free cluster enhancement (TFCE) method with family wise-error (FWE) correction for multiple comparisons [42]. The FA values of the brain regions showed abnormal white matter diffusion properties were extracted, averaged and correlated with the duration of migraine in patients group.

### Resting State Imaging Data Preprocessing

Data processing was performed using Analysis of Functional NeuroImages (AFNI) and FSL software according to previous studies [31], [33]. Preprocessing comprised the following steps: 1) remove first 5 volumes; 2) slice timing correction; 3) three-dimensional motion correction; 4) temporal despiking; 5) mean-based intensity normalization; 6) temporal band-pass filtering (0.0009–0.1 Hz); 7) linear and quadratic detrending; 8) nuisance signal removal (white matter, cerebrospinal fluid, global signal and 6 motion parameters) via multiple regression; 9) linear registration of functional to structural images (with intermediate registration to a low-resolution image and b_0_ unwarping); 10) nonlinear registration of structural images to the Montreal Neurological Institute (MNI) 152 template.

### Voxel-Mirrored Homotopic Connectivity

To account for geometric differences between hemispheres, we refined the registration from individual anatomic to MNI152 template space using the group-specific symmetrical template. In detail, all 42 registered structural images were averaged to create a mean image, which was then averaged with its left–right mirror to generate a group-specific symmetrical template using FSLUTILS. Nonlinear registration to this symmetrical template was performed for each participant, and the resultant transformation was applied to each participant’s preprocessed functional data. Homotopic RSFC was computed as the Pearson correlation (Fisher Z-transformed) between every pair of symmetrical inter-hemispheric voxels’ time series. The resultant correlations constitute VMHC. Since x = 0 defines the brain midline and there are no voxels medial of this plane, we excluded voxels medial of x = 0.

Global and regional group differences in VMHC were examined between the migraine and control group. Global VMHC was calculated by averaging VMHC values across all brain voxels within a unilateral hemispheric gray matter mask (there is only one correlation for each pair of homotopic voxels), which was created using the MNI152 gray matter tissue prior in FSL (threshold = 25% tissue-type probability). Group comparisons of global VMHC were performed using *t* tests. The significance threshold was *p*<0.05 and Bonferroni correction was used for multiple comparisons. With regard to the regional group differences in VMHC, the Permutation-based non-parametric testing with 10000 random permutations controlling for age and gender effects was employed. The threshold for significance was *p*<0.05, using TFCE method with FWE correction for multiple comparisons. The VMHC values of the brain regions showed abnormal inter-hemispheric connectivity were extracted, averaged and correlated with the duration of migraine in patients group.

### Seeding-Based RSFC

We examined the RSFC associated with areas exhibiting significantly different VMHC between groups. Specifically, we computed whole-brain voxelwise correlations associated with mean time series derived separately for two regions of interest (ROIs), comprising all voxels within the ACC area exhibiting reduced VMHC for migraine patients. Fisher Z–transformed correlation maps were then entered into a group-level voxelwise *t* test analysis including age and gender as covariates. Whole-brain correction for multiple comparisons was performed (*p*<0.05, FWE correction).

### Functional-Structural Relationship

To investigate how the inter-hemispheric RSFC are affected by the abnormalities of the CC, we assessed the relationship between the abnormal FA of CC and VMHC of the ACC in the migraine patients without aura. In detail, Pearson correlation was carried out between the FA value of the genu of CC and VMHC value of ACC in migraine patients.

## Results

### DTI Results

**Figure 1:**
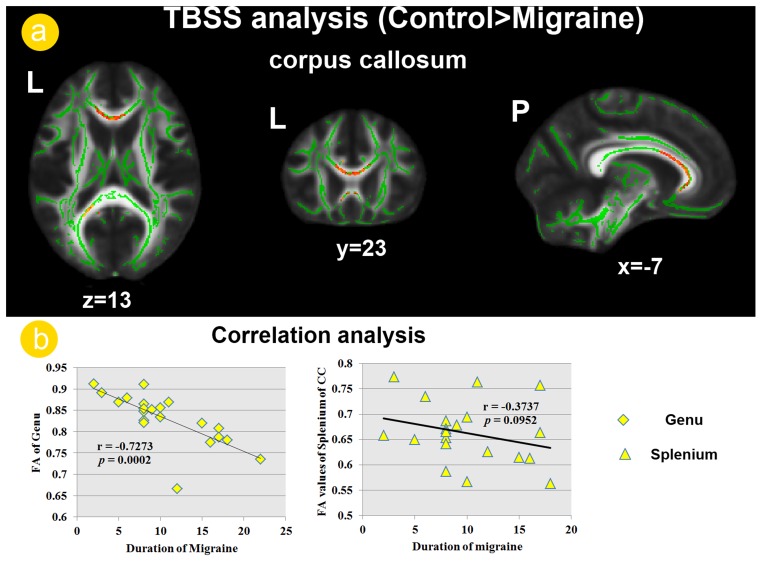
White matter abnormalities of the corpus callosum (CC) in migraine patients. a). Tract-based spatial statistics (TBSS) analysis revealed decreased fractional anisotropy (FA) values in the genu and the splenium of CC among patient group. b). the decreased FA values of the genu of CC were negatively correlated with duration of disease in migraine patients without aura.

In this study, we focused on the diffusion measurements of CC and only the findings of CC were reported here. TBSS analysis revealed that the migraine patients showed decreased FA values in the left splenium and bilateral genu of CC relative to the controls (Figure1. a). The correlation analysis revealed a significant negative correlation between the FA values of the genu of CC and duration of migraine in patients group (r = −0.7273; *p* = 0.0002), although the FA values of splenium of CC were not significant correlated with duration of migraine (r = −0.3737; *p* = 0.0952) (Figure1. b).

### Voxel-Mirrored Homotopic Connectivity

**Figure 2:**
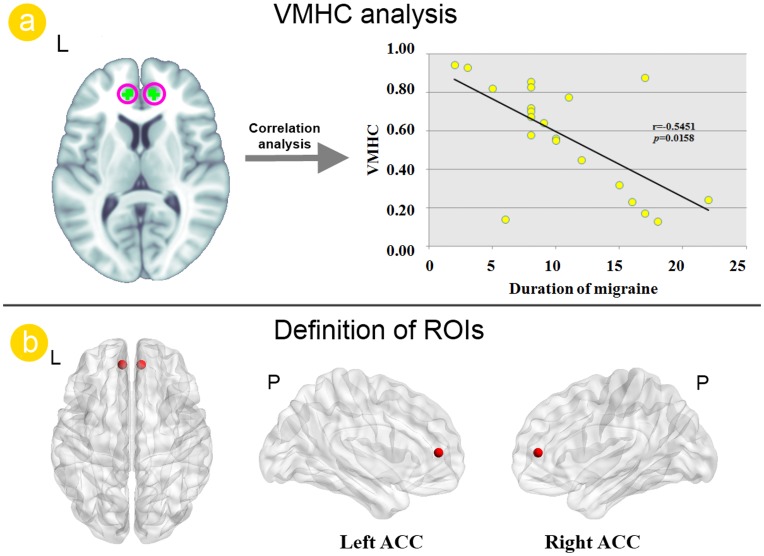
Voxel-mirrored homotopic connectivity (VMHC) analysis results between migraine patients and healthy controls. a). Resting state analysis revealed the reduced VMHC value of the ACC in migraine patients without aura. The VMHC value of the anterior cingulate cortex (ACC) was correlated with duration of migraine. b). The bilateral ACC were chosen as the region of interest (ROI) for subsequent seeding-based resting state functional connectivity (RSFC).

The global VMHC were not significant different (t(39) = 1.6501; *p* = 0.1149) between migraine (0.3794±0.0221) and control groups (0.4107±0.0236). However, the local comparisons revealed the ACC in which migraine exhibited reduced VMHC than controls after controlling for the age and sex (Figure2. a). No brain regions showed stronger VMHC in the migraine, relative to the control group. The correlation analysis indicated that the VMHC value of ACC negatively correlated with the duration of migraine in patients group (r = −0.5451; *p* = 0.0158) (Figure2. a).

### Seeding-Based RSFC

**Figure 3:**
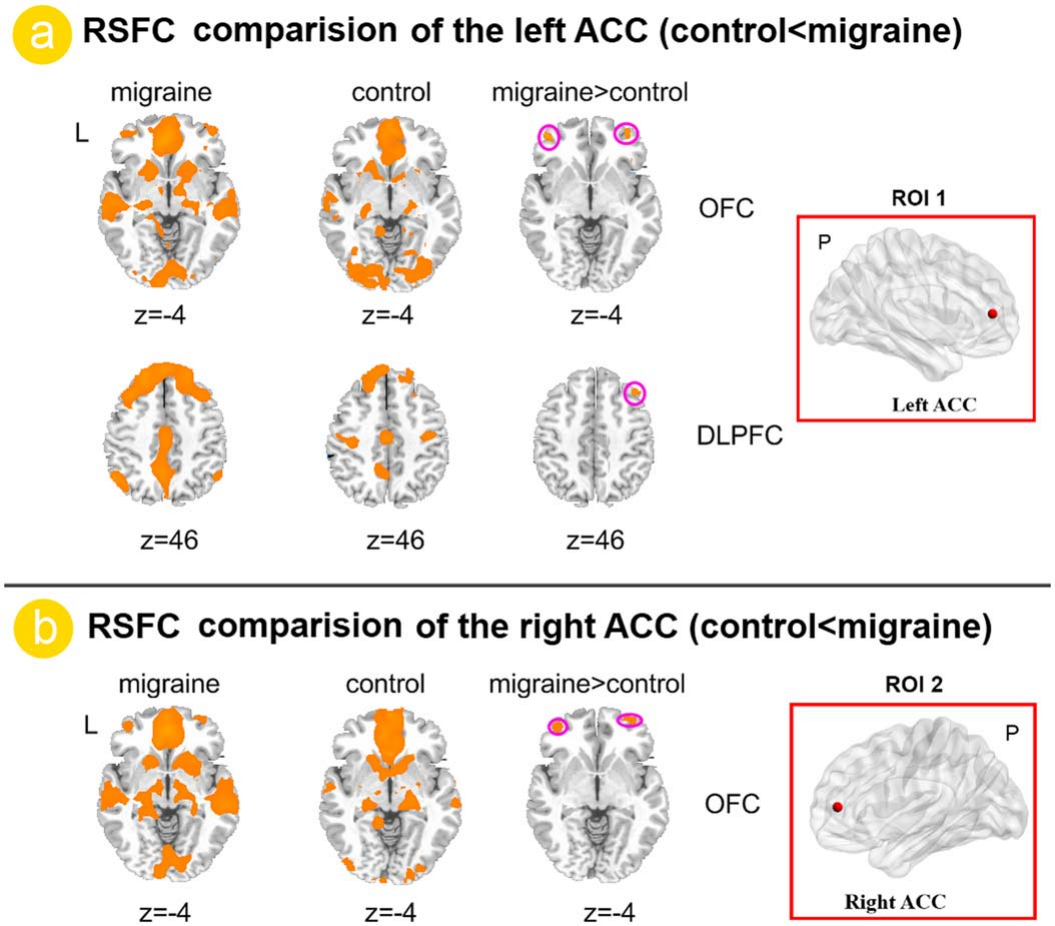
Functional connectivity analysis results between migraine patients and healthy controls. a). Relative to healthy controls, migraine patients without aura showed increased RSFC between the left anterior cingulate cortex (ACC) and the left dorsolateral prefrontal cortex (DLPFC) and right orbitofrontal cortex (OFC) (Figure3. a). With regards to the right ACC, the migraine patients without aura showed increased RSFC in the bilateral OFC.

We examined whole brain RSFC associated with the two ROIs (Figure1b), i.e. the left and right ACC, which exhibited reduced VMHC for migraine patients. Relative to healthy controls, migraine patients showed increased RSFC between the left ACC and the bilateral OFC and right dorsolateral prefrontal cortex (DLPFC) (Figure3. a). With regards to the right ACC, the migraine patients showed increased RSFC with the bilateral OFC (Figure3. b).

### Function-structure Relationship

**Figure 4:**
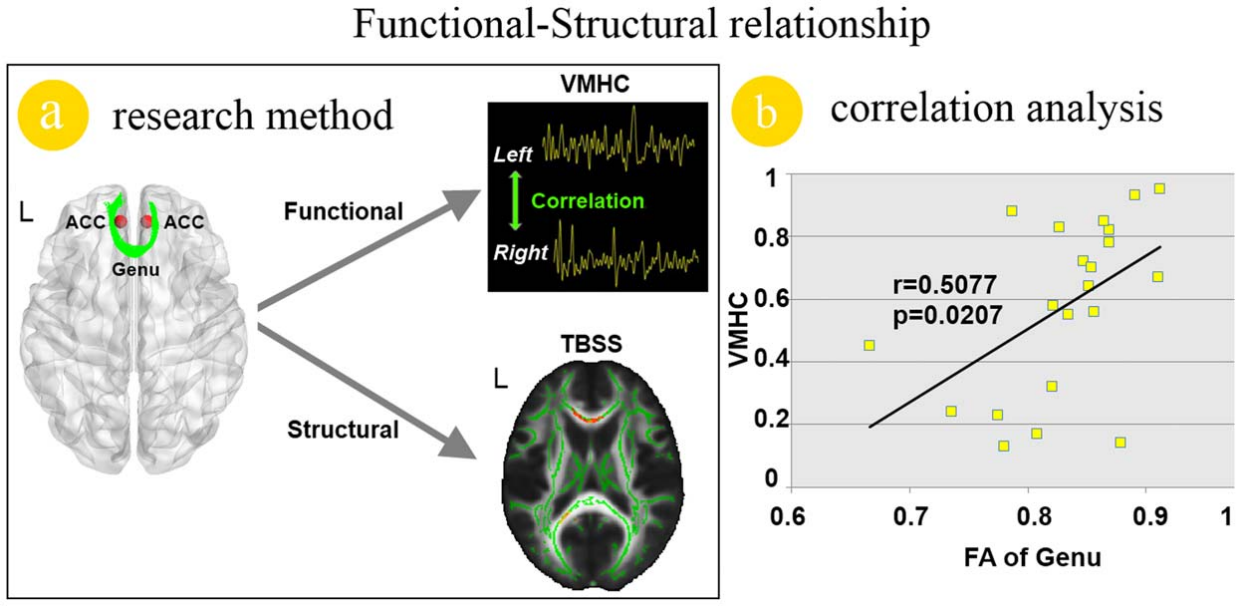
Functional and structural relationship analysis results in migraine patients. a). The demonstration of the methods in this study. b). The reduced voxel-mirrored homotopic connectivity (VMHC) value of the anterior cingulate cortex (ACC) was significant correlated with the decreased fractional anisotropy (FA) value of the genu of orpus callosum (CC) in migraine patients without aura.

To assess the effect of structural abnormalities of the genu of CC on inter-hemispheric RSFC of the ACC in migraine patients, the person correlation was introduced. Our results revealed a significant positive correlation (r = 0.5077; *p* = 0.0207) between the VMHC of the ACC and FA values of the genu of CC in the migraine patients (Figure4. b).

## Discussion

Migraine is the most common neurological disorder and cause significant individual and social burden due to chronic and unexpected pain attack [1]. In the past few decades, advanced neuroimaging had led to our evolution in our understanding of migraine pathophysiology [1], [2]. Brain structural and functional changes were both observed in patients with migraine and these alterations were negatively associated with longer migraine duration and attack frequency [1], [2], [7], [14], [15], [16]. Consistent with previous migraine study findings [17], our TBSS analysis revealed the genu and splenium part of CC showed decreased FA values in migraine patients without aura, relative to the healthy controls (Figure1. a). Furthermore, the reduced FA values of genu of CC were negatively correlated with the duration of disease (Figure1. b). As the largest white matter structure in the brain, the CC facilitates inter-hemispheric communication by providing the main route of communication between the two hemispheres of the brain [18], [19]. Abnormalities in white matter integrity in CC may influence inter-hemispheric RSFC. Therefore, we further investigated the inter-hemispheric RSFC between the migraine patients and healthy controls.

In the present study, we found reduced inter-hemispheric RSFC of ACC in migraine patients without aura (Figure2. a) and left ACC and right ACC networks both showed abnormal RSFC patterns (Figure3). The brain’s intrinsic functional architecture constitutes the foundation on which momentary neuronal responses underlying cognition and behavior are built [43], [44]. Thus, although we detected decrements in inter-hemispheric RSFC in ACC while participants were at rest, those decrements is likely to contribute to impaired function when that network is engaged in pain processing. As one of the most salient characteristics of the brain’s intrinsic functional architecture, homotopic functional connectivity can reflect the importance of inter-hemispheric communication to integrate brain function underlying coherent cognition and behavior [20], [30]. Altered inter-hemispheric functional interactions have been observed in several brain disorders [32], [33] and in normal aging [45], [46]. Our results provided further evidence of an association between migraine and disruptions between bilateral hemispheres, the inter-hemispheric communication of ACC especially (Figure2). Our findings suggested that attention should be paid to the roles of the disrupted functional circuitry as well.

Modern neuroimaging methods have explicated the neural substrate undying pain processing, namely pain matrix. It involves several cortical and subcortical brain regions, such as the thalamus, the amygdala, the insula cortex, the SMA, the posterior parietal cortex, the prefrontal cortex, the ACC, the periaqueductal gray, the basal ganglia, cerebellar cortex, the primary and secondary sensory cortex [15], [47]. As a key component of pain matrix, both structural and functional studies explicates ACC’s pivotal role in pain processing. Previous studies revealed that the affective responses of pain, such as unpleasantness, suffering and other negative effects, may be principally integrated in ACC [48], [49], [50], [51]. ACC is also involved in endogenous pain control, which is mediated by the endogenous opioid systems [52], [53]. The role of the ACC in migraine has been underpinned by recent neuroimaging studies. Positron emission tomography (PET) studies have shown an association between pain and abnormal activation of the ACC in migraine patients [5], [6]. Reduced regional homogeneity (ReHo) value of the BOLD signal in ACC was also observed in migraine patients without aura [14]. On the other hand, researchers using voxel-based morphormetry (VBM) method detected the decreased gray matter of ACC in migraine patients and significant correlations between the structural changes in ACC and disease duration [8], [9]. Therefore, we suggest that the VMHC abnormalities of ACC in migraine patients without aura (Figure2. a) validated the role of ACC in migraine.

Acknowledging that ACC dysfunction alone cannot explain the pathophysiology of migraine, therefore the bilateral ACC were chosen as the ROIs for whole brain RSFC analysis. Our results demonstrated that the left ACC showed increased RSFC with the right DLPFC and bilateral OFC (Figure3. a) and the right ACC showed increased RSFC with the bilateral OFC (Figure3. b) in migraine patients. The DLPFC has been shown to be involved with the cognitive dimension of pain processing, i.e. localization and encoding of the attended stimulus [54]. The OFC was involved in sensory integration, decision-making, expectation and planning behavior associated with sensitivity to reward and punishment [55]. All received sensations are modulated and related affective responses are assigned accordingly by the OFC [56], which is associated with the learning of the affective and motivational value of stimulation [57]. In addition, pain and pleasurable stimuli have been shown to elicit opioid release in the OFC [58], [59]. The increased functional connectivity between the ACC and DLPFC, between the ACC and OFC may explain the abnormal pain processing and executive function in migraine patients [12], [15], [60]. We suggested that frequent nociceptive input modified the frontal cortex resting state connectivity patterns and these changes may explain the functional impairments in migraine patients.

The correlation analysis demonstrated that the reduced inter-hemispheric RSFC of ACC in migraine patients without aura correlated with the duration of disease (Figure2. a), which suggested that longer duration of disease could result in more serious effects on the inter-hemispheric RSFC of ACC. Furthermore, the VMHC value in the ACC was positively correlated with the FA values of the genu of CC in the migraine patients (Figure4). Anatomically, the thinner axons in the genu connect the prefrontal cortex between the two hemispheres of the brain [18], [19]. The bilateral ACC communicated with one and the other by information transferring through the genu of CC (Figure4. a). Therefore, the white matter integrity abnormalities of the genu of CC probably affect the VMHC of ACC. Given that VMHC changes of the ACC were correlated with the FA changes of the genu of CC, we suggest that there is the possibility that the white matter changes of the genu of CC modulate the VMHC between bilateral ACC. In addition, previous studies had provided scientific evidence for that the RSFC can reflects structural connectivity in brain networks [61], [62], [63]. Our results demonstrated that the VMHC alterations of ACC can reflect the FA changes of the genu of CC in migraine patients without aura.

In the current study, it is worth noting that the VMHC changes were not randomly distributed, but concerned defined and highly pain related brain areas, i.e. ACC. It is indeed remarkable that the structural [1],[2],[15],[16] and functional [5], [6], [14] alterations seen in the ACC in migraine patients are similar to the changes reported in chronic back pain [64] and chronic phantom pain [65]. As most changes correlate to disease duration, it is plausible to suggest that the alteration of this region is a consequence, rather than a cause, of frequent nociceptive input. Our study used cross-sectional design and the question that whether these differences were consequence and precondition of migraine remains unclear. Although, correlation analysis indicated that the disease duration significant correlation with the reduced VMHC of the ACC (Figure2. a) and the reduced FA values of the genu of the CC (Figure1. b) in migraine patients without aura, this question could only be answered by investigating the temporal characteristics of experience-induced plasticity changes using a longitudinal design in the future. A comprehensive study design is also needed to investigate the exact relationship between the ACC functional changes during resting state and the CC structural changes.

In conclusion, we employed multimodal imaging methods to test the hypothesis that whether the abnormal white matter integrities of CC modulates inter-hemispheric RSFC in migraine patients without aura (Figure4. a). Our results demonstrated that the ACC showed reduced VMHC in migraine patients without aura and the inter-hemispheric RSFC changes of ACC correlated with the reduced FA value of the genu of the CC. Our findings provide further evidence of an association between the functional and structural alterations in migraine patients without aura. We suggested that the reduced FA value in genu of CC modulates inter-hemispheric RSFC in migraine patients without aura. It is hoped that combination of the functional and structural information may provide deeper insights into the migraine effects on the brain. However, more comprehensive experiment design with pain processing task are needed to investigate the accurate role of the VMHC abnormalities of ACC in migraine patients without aura.
